# Oxidative stress and endothelial function in normal pregnancy versus pre-eclampsia, a combined longitudinal and case control study

**DOI:** 10.1186/s12884-018-1685-5

**Published:** 2018-02-27

**Authors:** Dominique Mannaerts, Ellen Faes, Jan Gielis, Emeline Van Craenenbroeck, Paul Cos, Marc Spaanderman, Wilfried Gyselaers, Jerome Cornette, Yves Jacquemyn

**Affiliations:** 1 Departement of Obstetrics and Gynaecology, Antwerp University Hospital, Antwerp, Belgium; 2 ASTARC, Antwerp Surgical Training, Anatomy and Research Centre, University of Antwerp, Antwerp, Belgium; 3 Laboratory for Cellular and Molecular Cardiology and Department of Cardiology, Antwerp University Hospital, Edegem, Belgium; 4 Research Group Cardiovascular Diseases, Translational Pathophysiological Research, University of Antwerp, Antwerp, Belgium; 5 Laboratory for Microbiology, Parasitology and Hygiene (LMPH), University of Antwerp, Antwerp, Belgium; 6 Departement of Obstetrics and Gynecology, Maastricht University Medical Center, Maastricht, The Netherlands; 7 Departement of Obstetrics and Gynecology, Ziekenhuis-Oost-Limburg (ZOL), Genk, Belgium; 8 Departement of Obstetrics and Gynecology, Erasmus Medical Center, Rotterdam, The Netherlands; 9 Department of Obstetrics and Gynaecology, Antwerp Surgical Training and Anatomy Research Centre (ASTARC), Antwerp University/Antwerp University Hospital, Antwerp, Belgium

**Keywords:** Pre-eclampsia, Endothelial (dys) function, Oxidative stress, Electron paramagnetic resonance, Nitric oxide, Maternal outcome

## Abstract

**Background:**

Pre-eclampsia (PE) is related to an impaired endothelial function. Endothelial dysfunction accounts for altered vascular reactivity, activation of the coagulation cascade and loss of vascular integrity. Impaired endothelial function originates from production of inflammatory and cytotoxic factors by the ischemic placenta and results in systemic oxidative stress (OS) and an altered bioavailability of nitric oxide (•NO). The free radical •NO, is an endogenous endothelium-derived relaxing factor influencing endothelial function. In placental circulation, endothelial release of •NO dilates the fetal placental vascular bed, ensuring feto-maternal exchange. The Endopreg study was designed to evaluate in vivo endothelial function and to quantify in vitro OS in normal and pre-eclamptic pregnancies.

**Methods/design:**

The study is divided into two arms, a prospective longitudinal study and a matched case control study. In the longitudinal study, pregnant patients ≥18 years old with a singleton pregnancy will be followed throughout pregnancy and until 6 months post-partum. In the case control study, cases with PE will be compared to matched normotensive pregnant women. Maternal blood concentration of superoxide (O_2_•) and placental concentration of •NO will be determined using EPR (electron paramagnetic resonance). Endothelial function and arterial stiffness will be evaluated using respectively Peripheral Arterial Tonometry (PAT), Flow-Mediated Dilatation (FMD) and applanation tonometry. Placental expression of eNOS (endothelial NOS) will be determined using immune-histochemical staining. Target recruitment will be 110 patients for the longitudinal study and 90 patients in the case-control study.

**Discussion:**

The results of Endopreg will provide longitudinal information on in vivo endothelial function and in vitro OS during normal pregnancy and PE. Adoption of these vascular tests in clinical practice potentially predicts patients at risk to develop cardiovascular events later in life after PE pregnancies. •NO, O_2_•^−^ and eNOS measurements provide further inside in the pathophysiology of PE.

**Trial registration:**

This trial has been registered on clinicaltrials.gov. ClinicalTrials.gov Identifier: NCT02603913. Registered October 2015.

## Background

Pre-eclampsia (PE) is a potentially life-threatening pregnancy related vasculopathy characterized by hypertension and proteinuria. PE results in high morbi-mortality for both mother and her unborn child. Between five and 10 % of pregnancies are complicated by hypertensive disorders and worldwide the incidence of PE has increased by 25% in the past two decades [1].

In normal pregnancy vascular remodelling of the maternal uterine spiral arteries occurs. Trophoblast cells invade the spiral arterioles within the first 12 weeks of pregnancy and replace the muscular wall of the vessels converting them into wide bore, low resistance, large capacity vessels, a process normally completed by 20 weeks gestation [2]. The free radical nitric oxide (•NO) is an important mediator of the placentation process. •NO is an endogenous endothelium-derived relaxing factor influencing endothelial function. Under physiologic conditions, endothelial release of •NO in the placental circulation dilates the fetal placental vascular bed, ensuring feto-maternal exchange [3]. •NO is formed out of L-Arginine by NOS (Nitric Oxide Synthase). This reaction is regulated by VEGF (Vascular endothelial growth factor), an endothelial mitogen that has an important function in the proliferation of endothelial cells and in angiogenesis. VEGF stimulates eNOS (endothelial NOS) and induces therefore •NO production [4]. In an oxidative environment, the lack of NOS-stabilizing factors results in NOS-uncoupling. NOS-coupling causes a shift from •NO production to superoxide (O_2_•^−^) production which maintains an oxidative setting.

The pathogenesis of generalized endothelial dysfunction is well known in PE and is subdivided into two phases. The first phase exists of a poor trophoblast invasion of the spiral arteries during the placentation process, causing failure to transform the placental bed arteries from high to low resistance vessels. This results in local ischemia, reperfusion damage and oxidative stress (OS). The local damage activates the second phase where disturbed production of angiogenic and anti-angiogenic factors (placental growth factor (PlGF) and soluble fms-like tyrosine kinase 1 (sFlt-1), respectively) results in systemic inflammation, endothelial activation, systemic OS and altered endothelial •NO production [5, 6]. When this vascular endothelial activation and dysfunction occurs at the level of liver, kidney, brain and placenta, the clinical presentation of PE arises [7].

In the past, PE has been divided in two different entities; angiogenic or placental PE (formerly called early onset, before 34 weeks) and non-angiogenic or maternal PE (formerly called late onset, after 34 weeks) [8]. Impaired placentation and endothelial dysfunction have been described in placental PE, whereas pre-existing maternal cardiovascular risk factors (essential hypertension, high BMI, diabetes, renal disease,..) usually precede maternal PE. This description however oversimplifies and overstates recent existing findings. Maternal risk factors can precede early onset PE as well as abnormal concentrations of placental angiogenic factors are found in late onset PE [6]. Fetal growth restriction and endothelial dysfunction caused by systemic inflammation are usually described in placental PE, nevertheless they are common in late onset PE. It is therefore more accurate to state that both maternal and placental factors contribute to PE and research should focus on classifications based on pathophysiologic processes, for instance endothelial and vascular dysfunction and amount of systemic inflammation and OS [9, 10].

In normal pregnancy, placental OS is present during all three trimesters and is necessary to obtain normal cell function, including activation of redox-sensitive transcription factors and activation of protein kinases [11–15]. Although OS is a common necessary feature of normal pregnancy, persistent OS gives rise to different disease-states, such as PE [13, 15–17]. Although considerable research has been devoted to OS in PE [13–15], less attention has been paid to the evolution of OS during the course of normal pregnancy. Little research has described an increase in •NO concentration with gestational age, suggesting an important role for •NO in the cardiovascular changes of normal pregnancy [3].

Recent literature has elucidated that PE is an important risk factor for cardiovascular disease in later life. Bellamy et al. and McDonald et al. describe a 3-fold risk for hypertension and a 2-fold risk of ischemic heart disease and stroke in women with a history of PE [18–21]. Women with hypertensive disorders during pregnancy also have a greater risk of chronic kidney disease and end-stage renal disease [22, 23]. With a view to detecting those women at risk, objectifying endothelial function and vascular function after healthy pregnancy and PE can help to establish reference values for disturbed post-pregnancy vascular function.

## Methods/design

### Study hypothesis

Endothelial and vascular function improve during healthy pregnancy to answer the higher hemodynamic demands. Due to the deficient placentation in PE, disturbed production of (anti-) angiogenic and inflammatory factors results in arterial stiffness and endothelial dysfunction. After PE, this vascular dysfunction continues in patients at risk for developing cardiovascular events later in life.

A certain amount of OS is necessary in healthy pregnancy. The deficient placental oxygenation in PE causes excessive local formation of reactive oxygen and nitrogen species (O_2_•^−^ and •NO respectively). When the balance between pro-oxidant species and the antioxidants is disturbed, OS arises. We hypothesize that in PE, there is a higher amount of O_2_•^−^ in the maternal circulation and a lower concentration of •NO and eNOS measurable in the placenta.

Endothelial and vascular dysfunction is correlated to the amount of OS present in the circulation.

### Objectives

#### Primary study objective

To evaluate OS during pregnancy.

Measurement of •NO in placental tissue and O_2_•^−^ in maternal blood using EPR.

Measurement of eNOS in placental tissue using immuno-histochemical staining.

*Prospective longitudinal study*: To evaluate the OS profile in normal pregnancies.

*Matched case-control study:* To compare the OS profile in normal versus PE pregnancies.

#### Secondary study objectives

To evaluate endothelial function during pregnancy (using peripheral arterial tonometry (PAT) and flow mediated dilatation (FMD) techniques) and to relate endothelial function to •NO, O_2_•^−^ and eNOS concentration.

*Prospective longitudinal study:* To evaluate endothelial function in normal pregnancies.

*Matched case-control study:* To compare endothelial function in normal versus complicated pregnancies.

To evaluate arterial stiffness during pregnancy (Pulse wave velocity, pulse wave analysis using Sphygmocor ®) and to relate arterial stiffness to •NO, O_2_•^−^ and eNOS concentration.

*Prospective longitudinal study:* To evaluate arterial stiffness in normal pregnancies.

*Matched case-control study:* To compare arterial stiffness in normal versus complicated pregnancies.

### Methodology

#### Study design

##### Single centre prospective longitudinal study

The first part of the study will have a prospective longitudinal design. Pregnant women in their first trimester of pregnancy will be eligible and will be followed throughout pregnancy and until 6 months postpartum.

##### Multicentre matched case-control study

The second part is a case control study where patients with pregnancies complicated by PE will be compared to normotensive controls, matched for maternal and gestational age, parity, smoking behaviour, BMI and ethnic group. Patients will be followed throughout (the rest of their) pregnancy and until 6 months postpartum. Matching will reduce the risk of bias.

#### Study population

##### Inclusion criteria

1. Prospective longitudinal study

- Pregnant women ≥18 years old with a singleton pregnancy

2. Matched case-control study

- Pregnant women ≥18 years old with a singleton pregnancy and > 20 weeks of pregnancy
- Cases:

- Pre-eclampsia following the revised ISSHP definition (2014) is [24]:

- Hypertension (> 140 mmHg systolic or > 90 mmHg diastolic) developing after 20 weeks gestation and the coexistence of one or more of the following new onset conditions:

1. Proteinuria (spot urine protein/creatinine > 30 mg/mmol [0.3 mg/mg] or > 300 mg/day or at least 1 g/L [‘2 +’] on dipstick testing)
2. Other maternal organ dysfunction
  renal insufficiency (creatinine > 90 μmol/L)
  liver involvement (elevated transaminases – at least twice upper limit of normal - and/or severe right upper quadrant or epigastric pain)
  neurological complications (examples include eclampsia, altered mental status, blindness, stroke, or more commonly hyperreflexia when accompanied by clonus, severe headaches when accompanied by hyperreflexia, persistent visual scotomata)
  hematological complications (thrombocytopenia: platelet count below 150,000/dL, DIC, hemolysis)
3. Uteroplacental dysfunction: fetal growth restriction

- ◦ Hypertension greater than or equal to 140 mmHg systolic or 90 mmHg diastolic, must be confirmed at least 4 h apart. Hypertension greater than or equal to 160 mmHg systolic or 110 mmHg diastolic can be confirmed after a short interval (minutes) to facilitate antihypertensive treatment [25].

##### Exclusion criteria

Exclusion criteria are (gestational) diabetes, multiple pregnancies, fetal abnormalities, hypercholesterolemia, renal disease, auto-immune disorders and connective tissue disease. Intake of low-dose aspirin or vitamin C supplements (> 500 mg/day) is an exclusion criterion. Since in Belgium folic acid is an advised pre- and periconceptional therapy, this will not act as an exclusion criterion.

The use of other medication/supplements will be listed in the patients record to find eventual confounders afterwards.

#### Description of investigations (scheme of investigations, Fig. 1)

##### Endothelial function (FMD)

The gold standard for non-invasive assessment of endothelial function is FMD, measuring NO-dependent vasodilation of the brachial artery in response to reactive hyperaemia [26]. An ultrasound diagnostic instrument (Prosound alfa6, Hitachi Aloka Medical ®) equipped with vascular software for 2D-imaging, colour Doppler imaging and ECG-triggering, are used with a high frequency linear array transducer (UST-5413, 5-13 MHz, Hitachi Aloka Medical ®). Patients are in a resting, supine position with the arm in a comfortable position for imaging the brachial artery. A blood pressure cuff is placed on the forearm with the upper border of the cuff at a distance of 5 to 10 cm distal from the elbow (lateral epicondyle). The brachial artery is imaged above the antecubital fossa in a longitudinal plane with a clear delineation of both anterior and posterior intima-media interfaces. A special probe-holding device is used to ensure consistency of images during the measurement. The baseline artery diameter is automatically tracked and the waveform of diameter changes over the cardiac cycle is displayed in real time using an automated edge detection system. (eTracking system, Aloka ®). Arterial occlusion is created by cuff inflation to supra-systolic pressure at least 50 mmHg above systolic pressure (minimum value of 200 mmHg) for 5 min. Low–flow-mediated constriction (LFMC) is calculated as the percent decrease in arterial diameter in the last 30 s of cuff occlusion as compared with resting diameter [27]. After 5 min of occlusion, the cuff is deflated. Brachial diameter is recorded continuously (eTracking) from the time point of cuff inflation to 5 min after cuff deflation. FMD (in % from baseline value) is expressed as (post-ischemic maximal diastolic diameter change - baseline diastolic diameter)/baseline diastolic diameter [26]. During the measurements, brachial artery blood flows will be measured at 3 different time-points; at rest, 15 s before cuff deflation, and immediately upon cuff deflation using pulsed-wave Doppler [27]. All recordings are performed by two experienced investigators (IG, TS).

**Fig. 1:**
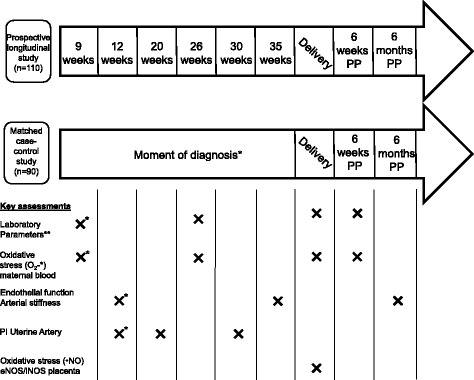
Study design and key assessments. Study design and data collection overview. PI = pulsatility index; PP = postpartum; * Assessments at moment of diagnosis; ** Laboratory parameters tested include NLR, MPV (mean platelet volume), platelet count, Fe, transferrin and ferritin

FMD and PAT measurements will be performed simultaneously and patients will be asked 24 h prior to examination not to eat high-fat substances nor drink caffeine or alcohol and to refrain from smoking at least 6 h prior to examination. Fingernails had to be short and no nail polish applied. Patients were studied in a quiet, temperature-controlled room (21-24 °C) and stressful situations for the patient were avoided (people entering the room unexpectedly, telephone ringtones, etc.) Patients will not by measured in active labour.

##### Endothelial function (RHI)

**Fig. 2:**
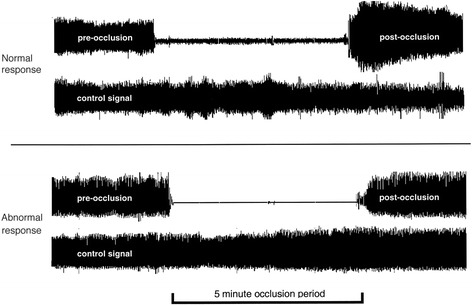
Representative reactive hyperaemia peripheral arterial tonometry recordings of subjects with normal and abnormal reactive hyperaemic response. Normal response is characterized by a distinct increase in the signal amplitude after cuff release compared with baseline

PAT is recorded using the Endo-PAT2000® (Itamar Medical, software version 3.2.4) and the disposable fingertip probes (Itamar Medical) in accordance with the manufacturer’s recommendations. PAT is a less operator-dependent and more reproducible technique. The system uses pneumatic finger probes which assess digital volume changes accompanying pulse waves. Reactive hyperaemia is induced as described for FMD and measurements are performed simultaneously to FMD. The ratio of the average amplitude of the PAT signal over a 1 min period starting 1 min after cuff deflation (maximum pulse amplitude) divided by the average amplitude of the PAT signal over a 3.5 min period before cuff inflation (baseline pulse amplitude) is calculated. The control arm is used to correct for confounding factors (room temperature, systemic changes). The result is expressed as the reactive hyperaemia index (RHI) (Fig. 2). All recordings are performed simultaneously with the FMD and by the same two experienced investigators (DM, EF).

##### Arterial stiffness: PWV, AP, AIx and AIx75

Pulse wave velocity and pulse wave analysis will be calculated using the Sphygmocor system ® (Atcor Medical, West Ryde, Australia). To calculate PWV, two pressure waveforms must be measured at a known distance apart and the distance between measurement sites is divided by the propagation time. Aortic PWV is measured by carotid-femoral PWV (cfPWV) as it is the ‘gold standard’ measurement of the stiffness of the aorta. Measurements of cfPWV will be performed using a pressure tonometer to transcutaneously record the pressure pulse waveform in the underlying artery. The tonometer contains a micromanometer that provides a very accurate recording of the pressure within the artery. The carotid and femoral PWV will be assessed by gently compressing respectively the carotid artery and the femoral artery with the tip of the tonometer at the site of maximal pulsation. The Sphygmocor device will automatically calculate the cfPWV.

Pulse wave analysis will calculate AIx by placing the tonometer at the radial artery (site of maximum pulsation). A generalized transfer function will derive the aortic pressure waveform from the radial artery waveform. From the aortic pressure waveform, the augmentation pressure (AP) and augmentation index (AIx) can be calculated. The AP (ΔP) is defined as the height of the late systolic peak above the inflection point on the waveform. The AIx is defined as AP expressed as a percentage of the aortic PP. As AIx is affected by heartrate, it will be standardized to a heart rate of 75 bmp (AIx-75).

##### Pulsatility index uterine artery and fetal biometry

Uterine artery Doppler examinations will be performed using trans-abdominal colour directed pulsed wave Doppler (Voluson, GE Healthcare Technologies, USA). Pulsatility index of both uterine arteries will be obtained on either side of the cervix before 14 weeks’ gestation and at the apparent crossover with the external iliac arteries after 14 weeks [28, 29].

Three similar consecutive waveforms must be obtained before calculating the PI. The mean PI of the two vessels was calculated. Observation on the presence or absence of a bilateral early protodiastolic notch will be performed. A notch is defined as a persistent decrease in blood flow velocity below the diastolic peak velocity, in early diastole.

At the same moment basic fetal biometry parameters will be measured: bi-parietal diameter, head circumference, abdominal circumference, femur length and expected fetal weight using Hadlocks formula [30].

##### Markers of systemic inflammation

Performing a complete blood count (CBC), the neutrophil/lymphocyte ratio (NLR), mean platelet volume (MPV) and platelet count will be obtained using a ADVIA 120 Hematology System (Siemens healthcare, Germany) [31–33].

##### Automated blood pressure measurement

SBP, DBP and MAP after 10 min rest in a sitting position, will be measured using a Mindray VS 900 monitor (Mindray ®, China).

#### Oxidative stress

##### Electron paramagnetic resonance

EPR (electron paramagnetic resonance) is derived from magnetic resonance spectroscopy and uses microwave radiation to detect molecules with an unpaired electron number, like radicals. When an magnetic field is created by the EPR spectrometer, all radicals will align. The EPR spectrometer sends out a radio frequent microwave, causing the electrons to jump from a low to a high energy state. This energy absorption can be measured and is directly correlated to the amount of free radicals in the sample. A ‘spin trap’ will be added to scavenge the very reactive radicals and to prolong their half live. EPR spectra will be obtained using the Bruker EMX 1273 spectrometer equipped with an ER 4119HS high-sensitivity resonator (=cavity) and 12-kW power supply operating at X band frequencies. The Bruker WINEPR -post processing system software (Germany, 1996) will be used to analyse the spectra [34].

##### Placental concentration of •NO

Placental tissue will be obtained within 2 min after (vaginal or caesarean) delivery. At a standardized central location, a viable sample of 1cm^3^ placental tissue will be taken avoiding placental infarcts. The sample will be rinsed with saline (NaCl) and immediately added to the spin trap 750 μl FeSO4 + 750 μl DETC (iron (II) diethyldithiocarbamate solution). After 1 h of incubation at 37 °C, the sample will be snap frozen and stored in − 80 °C until analysis with EPR [34].

Since labour causes ischemia-reperfusion injury at the site of the placenta, which will influence placental NO concentrations, samples will be subdivided in labour (vaginal delivery, secondary caesarean section) versus no labour (elective caesarean section) [35].

##### Maternal blood concentration of O_2_•^−^

Maternal blood will be obtained at 12 weeks and 24-28 weeks in a heparin tube (BD vacutainer) and transported on ice. After 15 min, 30 μl of spin trap CMH (1-hydroxy-3-methoxycarbonyl-2,2,5,5-tetramethylpyrrolidine) will be added to 30 μl blood. The whole will be incubated on ice and transferred into a capillary after 5 min. The sample will then be snap frozen and stored in − 80 °C until analysis [34].

##### Immuno-histochemical staining for eNOS and iNOS

Placental tissue will be obtained shortly after delivery and stained for eNOS and iNOS as previously described by Du et al. [36]

### Statistical methods

#### Sample size calculations

As sample size determining factor, we took the RHI since this is an important variable in our study, there is normal distribution and the population standard deviation is known.

##### Prospective longitudinal study

For the physiologic study of the RHI in pregnancy we calculate that for a 95% confidence interval, a population standard deviation of 0.5 (as described in other populations for RHI) and a tolerable standard error of the mean (SEM) value of 0.1, 97 women have to be followed [37]. Taking at least a 10% dropout into account the starting sample size will be 110 women.

##### Matched case-control study

In a pilot study by Yinon [38] the RHI in normotensive pregnancies was 1.8, and in PE 1.5; in most populations standard deviation is 0.5 [37]. For 80% power and a two sided α = 0.05 and considering a 0.3 difference clinically relevant, the sample size for each group would be 44 *(**http://www.stat.ubc.ca/~rollin/stats/ssize/n2.html**);* which we consider the sample size for our cross-sectional study comparing pre-eclamptic patients with normotensive controls. The sample size of this study will be 90 patients, 45 in each group.

#### Descriptive statistics and data analysis

##### Prospective longitudinal study

For the physiologic study of the RHI in pregnancy we will calculate the reference values and 95% confidence interval. Longitudinal data will be plotted and a linear mixed-effects model with random intercept will be fitted. Percentiles for RHI, FMD and PWV/PWA will be calculated based on this model. Correlation coefficients between baseline RHI, FMD PWV/PWA, UA Doppler PI, fetal biometry, NLR, MPV, MAP, birth-weight percentile, eNOS, •NO and O_2_•^−^ will be analysed.

##### Matched case-control study

RHI, FMD, PWV/PWA, UA Doppler PI, fetal biometry, NLR, MPV, MAP, birth-weight, eNOS, •NO and O_2_•^−^ concentration and other continuous variables in PE versus healthy pregnancies will be tested for normality using the Shapiro Wilk Test. If there is normality, they will be expressed as mean, standard deviations and 95% confidence intervals and compared using two sided T test. If not, they will be expressed as median and interquartile ranges and compared using Mann Whitney U Test.

## Discussion

PE is responsible for 11.5% of maternal deaths in Flanders. Ten percent of early neonatal deaths are caused by maternal hypertensive disorders [39]. This project will contribute to the knowledge of PE with the ultimate goal of reducing maternal morbidity and mortality.

As stated before, the gold standard for non-invasive assessment of endothelial function is FMD [26]. Previous literature suggests that during normal pregnancy, there is a steady increase in FMD until week 32, with a stabilization or even decline at week 36 [40]. In PE, a significant reduction in FMD is suggested [41]. Data using PAT during pregnancy and PE are limited, based on small studies and they show opposing results [38, 42]. Due to measurement of peripheral microcirculatory function, PAT is less NO-dependent than FMD. As such, FMD and PAT assess different aspects of vascular function [43–45]. Arterial stiffness has been evaluated in pregnancy, using applanation tonometry (AP) [2]. During normal pregnancy AIx falls during mid pregnancy and rises at the end of pregnancy. In PE AIx is significantly increased and a significant role of first trimester AIx in the early screening of PE has been proposed [2]. Arterial stiffness is independently associated with cardiovascular risk and may, therefore, provide a potential marker to select women who will develop cardiovascular events later in life after PE [2, 46]. Concerning endothelial function and arterial stiffness in normal pregnancy and PE there is still no consent in literature and further research is warranted.

Both in normal pregnancy and PE are inflammatory effects present, which can be objectified by higher neutrophil to lymphocyte ratio (NLR) and higher mean platelet volume (MPV) [47]. Increase in NLR and MPV are described to be more prominent in PE, and these factors have been proposed as predictive biomarkers for PE [31, 48]. Increased systemic low grade inflammation possibly contributes to alterations in endothelial function.

Previous research has demonstrated that markers of OS, like O_2_•^−^, hydrogen peroxide (H_2_O_2_), hydroxyl radical (•OH), nitric oxide (•NO), and peroxynitrite (ONOO^−^) are involved in the pathophysiology of placental pregnancy disorders. OS at the site of the placenta causes placental damage and this ischemic placenta releases cytotoxic, anti-angiogenic and inflammatory markers in the circulation resulting in systemic endothelial dysfunction and peripheral organ damage [3]. O_2_•^−^, the most abundant free radical, encloses an important role in the beneficial effects of OS. Studies measuring O_2_•^−^ concentration longitudinally in normal pregnancy and during PE are lacking, creating a gap in the knowledge of OS in pregnancy and PE. Evidence for OS at the placenta and in the maternal circulation in PE has led to the suggestion that anti-oxidant therapy can improve or even prevent PE. Vitamins E and C, L-arginine (precursor of •NO) and •NO donors have been proposed to limit both endothelial injury and for the prevention of PE with conflicting results [49–55]. Substantial knowledge of the evolution of OS in healthy and PE pregnancies might influence introduction of these therapies in regular medical practice.

ENDOPREG is the first clinical study comparing in vivo measurements of endothelial function with in vitro markers of endothelial dysfunction in a longitudinal and case-control setting. To our knowledge, serial changes in maternal endothelial function have not been evaluated previously in pregnancy using two different methods, i.e. PAT/FMD and EPR, simultaneously. The main strength of our study is the longitudinal design. Little studies determined maternal •NO or reactive oxygen species concentration at the moment of diagnosis [56] and only a few performed a longitudinal approach. Studying endothelial function and OS profile in and after normal pregnancies and PE, will give a better insight in the pathophysiology of this pregnancy complication and will help with the detection of patients a risk of developing cardiovascular events later in life. Within the ENDOPREG study, research groups from biomedical and pharmaceutical sciences will collaborate to unravel yet another step in the pathophysiology of the disease of many theories, PE.

## Abbreviations

•NO: Nitric oxide
AIx: Augmentation index
AP: Augmentation pressure
BMI: Body mass index
CBC: Complete blood count
Cf-PWV: Carotid-femoral pulse wave velocity
DBP: Diastolic blood pressure
EFW: Estimated fetal weight
eNOS: Endothelial nitric oxide synthase
EPR: Electron paramagnetic resonance
FMD: Flow mediated dilatation
FPR: False positive rate
IC: Informed consent
iNOS: Inducible nitric oxide synthase
IUGR: Intrauterine growth restriction
LFMC: Low–flow-mediated constriction
MAP: Mean arterial pressure
MPV: Mean platelet volume
NLR: Neutrophil to lymphocyte ratio
O_2_•^−^: Superoxide
ONOO-: Peroxynitrite
PAT: Peripheral artery tonometry
PE: Pre-eclampsia
PI: Pulsatility index
PlGF: Placental growth factor
PP: Pulse pressure
PWV: Pulse wave velocity
RHI: Reactive hyperemia index
RNS: Reactive nitrogen species
ROS: Reactive oxygen species
SBP: Systolic blood pressure
SEM: Standard error of the mean
UA: Uterine artery
VEGF: Vascular endothelial growth factor
